# The digital transformation and future era: bibliometric view of artificial intelligence application in pediatric surgery

**DOI:** 10.3389/fped.2025.1528666

**Published:** 2025-06-12

**Authors:** Boshen Shu, Shufeng Zhang, Jian Gao, Lin Wang, Xiaohui Wang

**Affiliations:** Department of Pediatric Surgery, Henan Provincial People’s Hospital, Zhengzhou, Henan, China

**Keywords:** artificial intelligence, AI, pediatric surgery, bibliometrics, visualized study

## Abstract

**Introduction:**

Artificial intelligence has been extensively used in the personalized diagnosis and treatment of pediatric surgery. Numerous articles have been published related to this research recently. Consequently, we aimed to perform a bibliometric analysis of influential studies to reveal the digital transformation and future era within pediatric surgery.

**Methods:**

We searched publications on artificial intelligence application in pediatric surgery until December 31, 2023, via Web of Science core collection database comprehensively. Of these, the 100 most cited articles were evaluated in detail. Diverse parameters including total citations, publication year, journal, impact factor, impact index, country, organization, keyword, study design and evidence level were analyzed. Bibliometrix package from Rstudio, VOSviewer and GraphPad Prism were used for data analysis and mapping.

**Results:**

A total of 2,799 publications were searched and the 100 most cited articles were published from 1995 to 2023, with a total citation number of 2,770. The top country and organization contributing to this area were the USA and Stanford University, while the *Journal of Pediatric Surgery* dominated the number of studies from the top 100. Retrospective study and articles with evidence level III were the most common. For keyword co-occurrence analysis, it indicated necrotizing enterocolitis, congenital heart disease and radiomics dominated potential hotspots in the future.

**Conclusions:**

The present study presents a detailed list of the impactful articles on artificial intelligence application in pediatric surgery. It provides insights into potential cooperation and prospects for future research, which plays a helpful reference for researchers studying on artificial intelligence application in pediatric surgery.

## 1 Introduction

Artificial intelligence (AI) mainly refers to the utilization of computers or machines to simulate human intelligent behavior, including learning, cognitive functions, problem-solving, perception and many other characteristics (1). Since McCarthy et al. mentioned the AI conception in the 1950s, it has progressively evolved to be a multidisciplinary subject (2). Incorporating various areas such as computing, mathematics, biology, mechanical engineering and several more sectors. Due to the powerful potential of its advanced algorithms and learning capabilities, AI has demonstrated significant application in different medical domains like cardiovascular disease and rheumatic disease (3, 4). It now shows considerable reliability in disease diagnosis, prognosis prediction, drug research, as well as other fields (5, 6).

Wide applications of AI hold substantial potential to transform child and adolescent health digitally. The special challenges related to children, containing distinctive developmental and physiological requirements, heterogeneous cognitive capabilities, and natural communication problems, emphasize the revolutionary potential of AI in this area (7). For pediatric surgery, precise diagnosis, timely predictions, patient's safety and therapy strategies can be remarkably reinforced by the combination with AI in patient care process (8). Notably, numerous articles were published related to AI application in pediatric surgery recently. Consequently, it is of crucial importance for scholars to grasp the latest studies for reviewing the substantial update. Bibliometric analysis is a type of statistical approach that takes citation counts as a main measure of research influence, serving as a useful tool to evaluate development tendencies of a certain research field (9). This method has been applied in many different medical research fields to depict the knowledge structure and development trends till today (10, 11). However, to the best of our knowledge, there is no research on bibliometric analysis for artificial intelligence application in pediatric surgery. Hence, we aimed to perform a bibliometric analysis to explore the current research topics and cooperative networks in the application of AI in pediatric surgery over recent years, for the purpose of providing a theoretical reference for scholars to better seize the research frontiers and future trends.

## 2 Materials and methods

### 2.1 Data collection and search strategy

In October 2024, we performed a literature search in Web of Science Core Collection (WoSCC). A comprehensive review of pertinent studies was taken to support the formulation for our search strategy. Besides, for obtaining only related search results, a “title” instead of “topic” searching strategy was used (12, 13). Concerning the application of AI in pediatric surgery, we conducted the following searching terms: (“artificial intelligence” OR “computational intelligence” OR “machine learning” OR “deep learning” OR “decision trees” OR “decision forest” OR “expert system” OR “fuzzy logic” OR “automatic programming” OR “autonomous robot” OR “intelligent agent” OR “neural net” OR “voice recognition” OR “text mining” OR “electronic health record” OR “AI” OR “ML” OR “SVM” OR “Random forest” OR “Logistic regression” OR “RNN” OR “LSTM”) (Title) AND (“neonate” OR “neonates” OR “neonatal” OR “infant” OR “infants” OR “infancy” OR “preterm” OR “preterms” OR “newborn” OR “newborns” OR “pediatric” OR “pediatrics” OR “children” OR “child” OR “boy” OR “girl” OR “boys” OR “girls” OR “adolescent” OR “congenital” OR “atresia” OR “tracheoesophageal fistula” OR “necrotizing enterocolitis” OR “Hirschsprung disease” OR “anorectal malformation” OR “neuroblastoma” OR “hepatoblastoma” OR “nephroblastoma” OR “wilms” OR “orchidopexy” OR “pyloromyotomy” OR “Kasai” OR “imperforate anus” OR “pediatric surgery”) (Title) AND LA = (English) AND Publication time span = (1 January 1945 to 31 December 2023).

### 2.2 Data extraction and including criteria

The included articles were restricted to those that (1) involved AI applications, (2) and were relevant to pediatric surgery field (3) were published as article or review. Two independent authors screened all the publications by reading abstracts or full text according to the criteria. Differences between these two authors were resolved through thorough discussion. The selected articles based on the unanimous decision from these two reviewers were ranked in a descending order and the first 100 came to be the final list.

The following factors of the 100 most cited manuscripts were recorded and analyzed: total citations, publication year, journal, impact factor (IF), impact index, country, organization, keyword, study design and evidence level. The impact index is calculated by dividing the duration time (unit: year) since publication by the number of cited times, multiplied by 100, with lower outcome demonstrating a more robust impact (14). The study designs included retrospective study, prospective study, review, case-control study, randomized controlled trial (RCT), meta-analysis and systematic review. The evidence levels were arranged in accordance with Cashin et al. from high to low: meta-analysis (Level I), RCT (Level I), systematic review (Level I), prospective study (Level II), retrospective study (Level III), review (Level IV) and case-control study (Level IV) (15). Level I and II were defined as high evidence levels.

### 2.3 Data analysis and visualization

Statistical analyses were conducted with GraphPad Prism v. 7.0 (GraphPad, La Jolla, CA, USA). All tests were two-sided. Spearman correlation coefficient was applied to examine correlations among selected continuous variables. Unpaired *t* tests were taken to make the comparison between two different groups for parametric data and the One-way ANOVA test was performed for non-parametric data. The *P* Value of <0.05 was considered statistically significant.

Visualized analysis for country and organization collaboration, as well as keyword co-occurrence network analysis were conducted by VOSviewer 1.6.20 (Leiden University, Leiden, The Netherlands). Here, the line thickness between the colored nodes represents the total link strength. While for bibliometrix package from Rstudio, the “Most Relevant Sources” function was applied to describe the rank of publications from different journals. The “Most Relevant Affiliations” function was used to identify the contribution from different organizations.

## 3 Results

### 3.1 Overview

**Figure 1:**
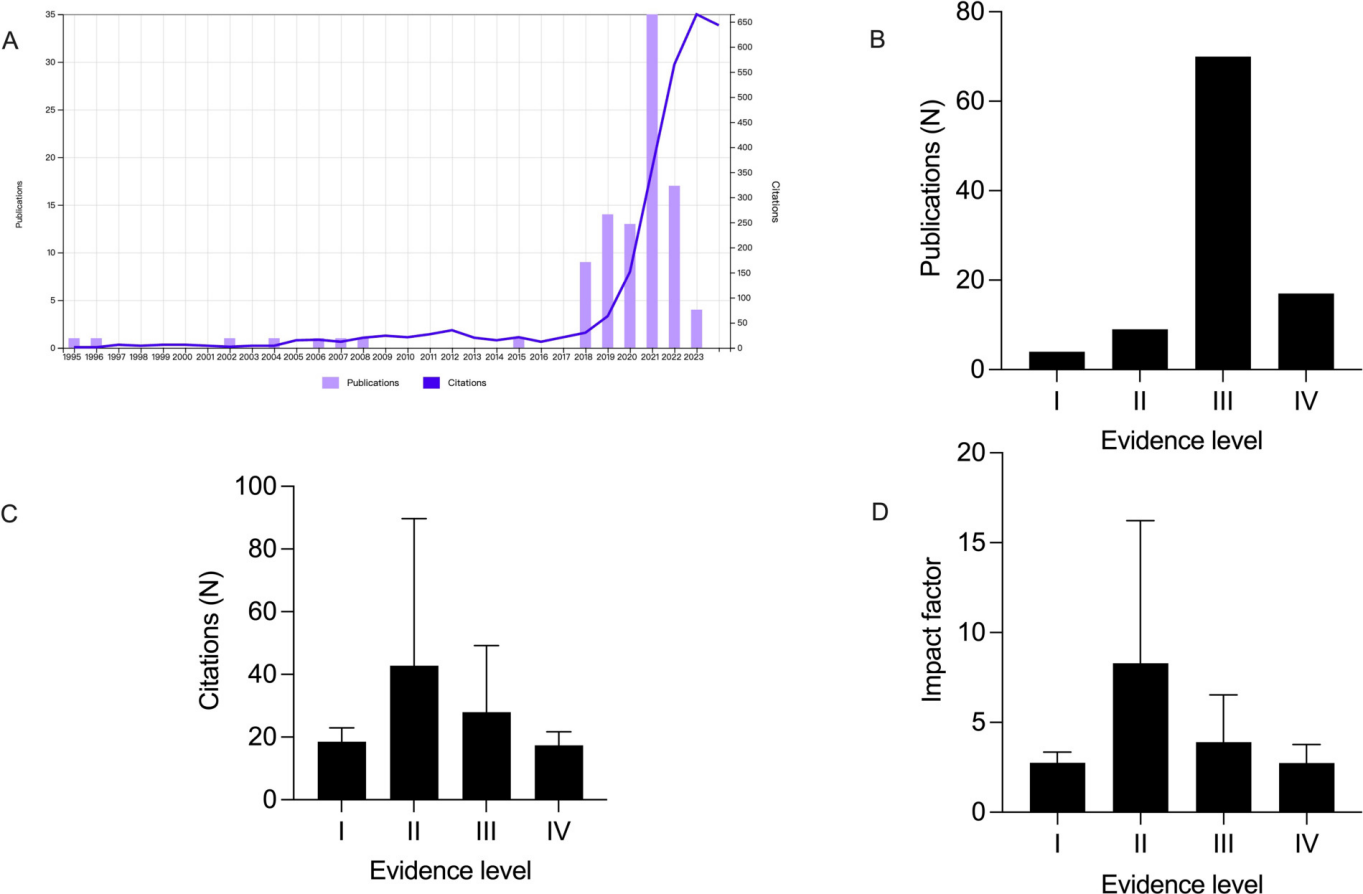
**(A)** Trends in the number of top 100 cited articles and their citations. **(B)** The evidence level distribution of top 100 cited publications. **(C)** Mean citation number per publication according to the evidence level. **(D)** Mean impact factor per corresponding journal according to the evidence level. No significant difference was found in citations per publication among different evidence levels (*P* = 0.057); no significant difference was found in impact factor per corresponding journal among different evidence levels (*P* = 0.095).

**Figure 2:**
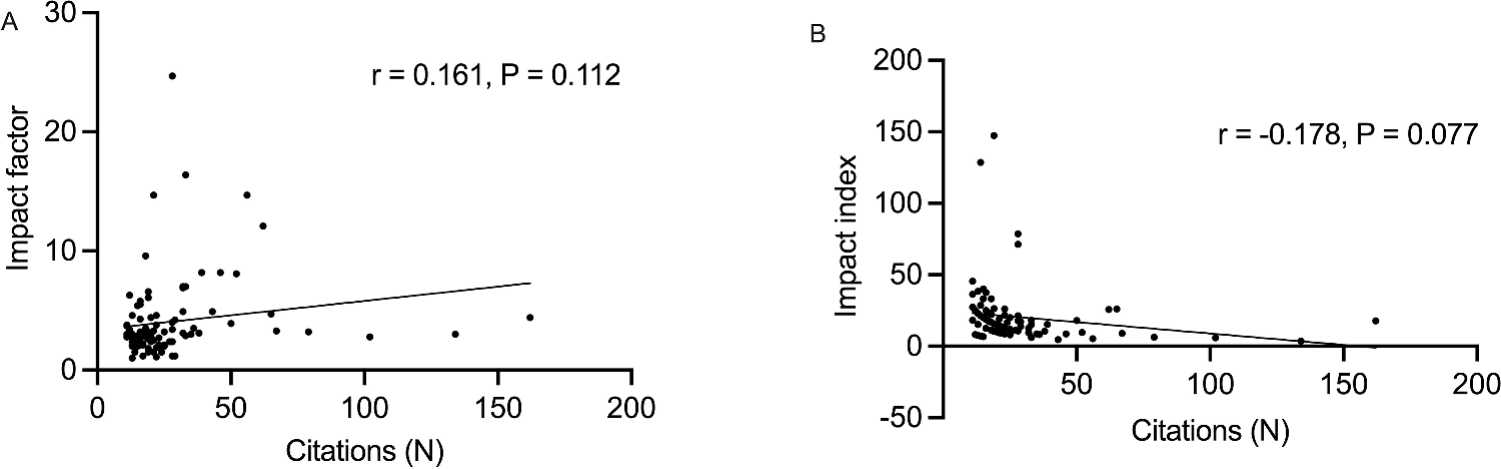
**(A)** The correlation between the number of citations and impact factor for the top 100 cited publications. **(B)** The correlation between the number of citations and impact index for the top 100 cited publications. There were no correlations between the number of citations and impact factor or impact index, respectively, for the top 100 cited articles.

A total of 2,799 manuscripts were identified by the initial search. The top 100 cited articles were published between 1995 and 2023 and they were presented accordingly in Table 1. The total number of citations was 2,773 (2,710 without self-citations; range from 11 to 162). The number of publications and citations each year generally increased from 1995 to 2023 and the year 2021 held the leading position in the number of publications (*n* = 35) (Figure 1A). The most cited article entitled “Prediction of progression of the curve in girls who have adolescent idiopathic scoliosis of moderate severity. Logistic regression analysis based on data from The Brace Study of the Scoliosis Research Society” was published in 1995 by Peterson et al. in *Journal of Bone and Joint Surgery-American Volume* (16). While the article with the lowest impact index entiteled “Real-time cardiovascular MR with spatio-temporal artifact suppression using deep learning-proof of concept in congenital heart disease” was published in 2019 by Hauptmann A et al. in the journal *Magnetic Resonance in Medicine* (17). There were 75 retrospective studies, 9 prospective studies, 9 reviews, 3 case-control studies, 2 RCTs, 1 meta-analysis and 1 systematic review papers on the top 100 list. Articles with evidence level III dominated the leading position (*n* = 75), followed by level IV (*n* = 12) and level II (*n* = 9) (Figure 1B). There was no significant difference between evidence level with either citation number per article (*P* = 0.057) (Figure 1C) or impact factor of the corresponding journal (*P* = 0.095) (Figure 1D). The number of cited times did not correlate with the IF (*r* = 0.161, *P* = 0.112) (Figure 2A) or II (*r* = −0.178, *P* = 0.077) (Figure 2B) per article significantly.

**Table 1:** The top 100 cited articles on AI application in pediatric surgery.

Rank	Author	Title	Journal	Citations (N)	Year	Impact factor	Impact index
1	Peterson et al.	Prediction of progression of the curve in girls who have adolescent idiopathic scoliosis of moderate severity. Logistic regression analysis based on data from The Brace Study of the Scoliosis Research Society	JOURNAL OF BONE AND JOINT SURGERY-AMERICAN VOLUME	162	1995	4.4	17.9
2	Hauptmann et al.	Real-time cardiovascular MR with spatio-temporal artifact suppression using deep learning-proof of concept in congenital heart disease	MAGNETIC RESONANCE IN MEDICINE	134	2019	3	3.7
3	Zhang et al.	Deep Learning-Based Multi-Omics Data Integration Reveals Two Prognostic Subtypes in High-Risk Neuroblastoma	FRONTIERS IN GENETICS	102	2018	2.8	5.9
4	Ladefoged et al.	Deep Learning Based Attenuation Correction of PET/MRI in Pediatric Brain Tumor Patients: Evaluation in a Clinical Setting	FRONTIERS IN NEUROSCIENCE	79	2019	3.2	6.3
5	Hale et al.	Machine-learning analysis outperforms conventional statistical models and CT classification systems in predicting 6-month outcomes in pediatric patients sustaining traumatic brain injury	NEUROSURGICAL FOCUS	67	2018	3.3	9.0
6	Neff et al.	Prediction of mortality and need for neonatal extracorporeal membrane oxygenation in fetuses with congenital diaphragmatic hernia: Logistic regression analysis based on MRI fetal lung volume measurements	AMERICAN JOURNAL OF ROENTGENOLOGY	65	2007	4.7	26.2
7	Büsing et al.	MR lung volume in fetal congenital diaphragmatic hernia: Logistic regression analysis–Mortality and extracorporeal membrane oxygenation	RADIOLOGY	62	2008	12.1	25.8
8	Zhou et al.	Ensembled deep learning model outperforms human experts in diagnosing biliary atresia from sonographic gallbladder images	NATURE COMMUNICATIONS	56	2021	14.7	5.4
9	Rayan et al.	Binomial Classification of Pediatric Elbow Fractures Using a Deep Learning Multiview Approach Emulating Radiologist Decision Making	RADIOLOGY-ARTIFICIAL INTELLIGENCE	52	2019	8.1	9.6
10	Chong et al.	Predictive modeling in pediatric traumatic brain injury using machine learning	BMC MEDICAL RESEARCH METHODOLOGY	50	2015	3.9	18.0
11	Xiao et al.	Follow the Sound of Children's Heart: A Deep-Learning-Based Computer-Aided Pediatric CHDs Diagnosis System	IEEE INTERNET OF THINGS JOURNAL	46	2020	8.2	8.7
12	Dupuis et al.	External validation of a commercially available deep learning algorithm for fracture detection in children	DIAGNOSTIC AND INTERVENTIONAL IMAGING	43	2022	4.9	4.7
13	Ren et al.	Maternal exposure to ambient PM10 during pregnancy increases the risk of congenital heart defects: Evidence from machine learning models	SCIENCE OF THE TOTAL ENVIRONMENT	39	2018	8.2	15.4
14	Quon et al.	Deep Learning for Pediatric Posterior Fossa Tumor Detection and Classification: A Multi-Institutional Study	AMERICAN JOURNAL OF NEURORADIOLOGY	38	2020	3.1	10.5
15	Chen et al.	CT-Based Radiomics Signature With Machine Learning Predicts MYCN Amplification in Pediatric Abdominal Neuroblastoma	FRONTIERS IN ONCOLOGY	36	2021	3.5	8.3
16	Attallah	MB-AI-His: Histopathological Diagnosis of Pediatric Medulloblastoma and its Subtypes via AI	DIAGNOSTICS	35	2021	3	8.6
17	Peng et al.	Deep learning-based automatic tumor burden assessment of pediatric high-grade gliomas, medulloblastomas, and other leptomeningeal seeding tumors	NEURO-ONCOLOGY	33	2022	16.4	6.1
18	Reismann et al.	Diagnosis and classification of pediatric acute appendicitis by artificial intelligence methods: An investigator-independent approach	PLOS ONE	33	2019	2.9	15.2
19	García-Cano et al.	Prediction of spinal curve progression in Adolescent Idiopathic Scoliosis using Random Forest regression	COMPUTERS IN BIOLOGY AND MEDICINE	33	2018	7	18.2
20	Salekin et al.	Multimodal spatio-temporal deep learning approach for neonatal postoperative pain assessment	COMPUTERS IN BIOLOGY AND MEDICINE	32	2021	7	9.4
21	Tajdari et al.	Image-based modelling for Adolescent Idiopathic Scoliosis: Mechanistic machine learning analysis and prediction	COMPUTER METHODS IN APPLIED MECHANICS AND ENGINEERING	32	2021	6.9	9.4
22	Zhang et al.	Clinical application of artificial intelligence-assisted diagnosis using anteroposterior pelvic radiographs in children with developmental dysplasia of the hip	BONE & JOINT JOURNAL	32	2020	4.9	12.5
23	Zhou et al.	Automatic Machine Learning to Differentiate Pediatric Posterior Fossa Tumors on Routine MR Imaging	AMERICAN JOURNAL OF NEURORADIOLOGY	32	2020	3.1	12.5
24	Karimi-Bidhendi et al.	Fully-automated deep-learning segmentation of pediatric cardiovascular magnetic resonance of patients with complex congenital heart diseases	JOURNAL OF CARDIOVASCULAR MAGNETIC RESONANCE	29	2020	4.2	13.8
25	Wadhwani et al.	Predicting ideal outcome after pediatric liver transplantation: An exploratory study using machine learning analyses to leverage Studies of Pediatric Liver Transplantation Data	PEDIATRIC TRANSPLANTATION	29	2019	1.2	17.2
26	Nurmaini et al.	Deep Learning-Based Computer-Aided Fetal Echocardiography: Application to Heart Standard View Segmentation for Congenital Heart Defects Detection	SENSORS	28	2021	3.4	10.7
27	Lv et al.	Artificial intelligence-assisted auscultation in detecting congenital heart disease	EUROPEAN HEART JOURNAL—DIGITAL HEALTH	28	2021	4	10.7
28	Bertsimas et al.	Comparison of Machine Learning Optimal Classification Trees With the Pediatric Emergency Care Applied Research Network Head Trauma Decision Rules	JAMA PEDIATRICS	28	2019	24.7	17.9
29	Irles et al.	Estimation of Neonatal Intestinal Perforation Associated with Necrotizing Enterocolitis by Machine Learning Reveals New Key Factors	INTERNATIONAL JOURNAL OF ENVIRONMENTAL RESEARCH AND PUBLIC HEALTH	28	2018	NA	21.4
30	Zheng et al.	The impact of pharmacogenomic factors on steroid dependency in pediatric heart transplant patients using logistic regression analysis	PEDIATRIC TRANSPLANTATION	28	2004	1.2	71.4
31	DiRusso et al.	Development of a model for prediction of survival in pediatric trauma patients: Comparison of artificial neural networks and logistic regression	JOURNAL OF PEDIATRIC SURGERY	28	2002	2.4	78.6
32	Lure et al.	Using machine learning analysis to assist in differentiating between necrotizing enterocolitis and spontaneous intestinal perforation: A novel predictive analytic tool	JOURNAL OF PEDIATRIC SURGERY	27	2021	2.4	11.1
33	Attallah et al.	AI-Based Pipeline for Classifying Pediatric Medulloblastoma Using Histopathological and Textural Images	LIFE-BASEL	25	2022	3.2	8.0
34	Marcinkevics et al.	Using Machine Learning to Predict the Diagnosis, Management and Severity of Pediatric Appendicitis	FRONTIERS IN PEDIATRICS	25	2021	2.1	12.0
35	Arafati et al.	Artificial intelligence in pediatric and adult congenital cardiac MRI: an unmet clinical need	CARDIOVASCULAR DIAGNOSIS AND THERAPY	25	2019	2	20.0
36	Aydin et al.	A novel and simple machine learning algorithm for preoperative diagnosis of acute appendicitis in children	PEDIATRIC SURGERY INTERNATIONAL	24	2020	1.5	16.7
37	Huang et al.	Artificial Intelligence Applications in Pediatric Brain Tumor Imaging: A Systematic Review	WORLD NEUROSURGERY	23	2022	1.9	8.7
38	Quon et al.	Artificial intelligence for automatic cerebral ventricle segmentation and volume calculation: a clinical tool for the evaluation of pediatric hydrocephalus	JOURNAL OF NEUROSURGERY-PEDIATRICS	23	2021	2.1	13.0
39	Daldrup-Link	Artificial intelligence applications for pediatric oncology imaging	PEDIATRIC RADIOLOGY	23	2019	2.1	21.7
40	Bartz-Kurycki et al.	Enhanced neonatal surgical site infection prediction model utilizing statistically and clinically significant variables in combination with a machine learning algorithm	AMERICAN JOURNAL OF SURGERY	23	2018	2.7	26.1
41	Hayashi et al.	Automated detection of acute appendicular skeletal fractures in pediatric patients using deep learning	SKELETAL RADIOLOGY	22	2022	1.9	9.1
42	Zeng et al.	Explainable machine-learning predictions for complications after pediatric congenital heart surgery	SCIENTIFIC REPORTS	22	2021	3.8	13.6
43	Bertsimas et al.	Adverse Outcomes Prediction for Congenital Heart Surgery: A Machine Learning Approach	WORLD JOURNAL FOR PEDIATRIC AND CONGENITAL HEART SURGERY	22	2021	1.1	13.6
44	Jalali et al.	Machine Learning Applied to Registry Data: Development of a Patient-Specific Prediction Model for Blood Transfusion Requirements During Craniofacial Surgery Using the Pediatric Craniofacial Perioperative Registry Dataset	ANESTHESIA AND ANALGESIA	22	2021	4.6	13.6
45	Truong et al.	Application of machine learning in screening for congenital heart diseases using fetal echocardiography	INTERNATIONAL JOURNAL OF CARDIOVASCULAR IMAGING	21	2022	1.5	9.5
46	Tunthanathip et al.	Comparison of intracranial injury predictability between machine learning algorithms and the nomogram in pediatric traumatic brain injury	NEUROSURGICAL FOCUS	21	2021	3.3	14.3
47	Killian et al.	Machine learning-based prediction of health outcomes in pediatric organ transplantation recipients	JAMIA OPEN	21	2021	2.5	14.3
48	Toba et al.	Prediction of Pulmonary to Systemic Flow Ratio in Patients With Congenital Heart Disease Using Deep Learning-Based Analysis of Chest Radiographs	JAMA CARDIOLOGY	21	2020	14.7	19.0
49	Choi et al.	Deep Learning-Assisted Diagnosis of Pediatric Skull Fractures on Plain Radiographs	KOREAN JOURNAL OF RADIOLOGY	20	2022	4.4	10.0
50	Mullen et al.	Race and Genetics in Congenital Heart Disease: Application of iPSCs, Omics, and Machine Learning Technologies	FRONTIERS IN CARDIOVASCULAR MEDICINE	20	2021	2.8	15.0
51	Day et al.	Artificial intelligence, fetal echocardiography, and congenital heart disease	PRENATAL DIAGNOSIS	20	2021	2.7	15.0
52	Dhaliwal et al.	Accurate Classification of Pediatric Colonic Inflammatory Bowel Disease Subtype Using a Random Forest Machine Learning Classifier	JOURNAL OF PEDIATRIC GASTROENTEROLOGY AND NUTRITION	20	2021	2.4	15.0
53	Lin et al.	Interpretable prediction of necrotizing enterocolitis from machine learning analysis of premature infant stool microbiota	BMC BIOINFORMATICS	19	2022	2.9	10.5
54	Liu et al.	Deep learning-based computer-aided heart sound analysis in children with left-to-right shunt congenital heart disease	INTERNATIONAL JOURNAL OF CARDIOLOGY	19	2022	3.2	10.5
55	Shi et al.	Explainable machine learning model for predicting the occurrence of postoperative malnutrition in children with congenital heart disease	CLINICAL NUTRITION	19	2022	6.6	10.5
56	Tunthanathip et al.	Application of machine learning to predict the outcome of pediatric traumatic brain injury	CHINESE JOURNAL OF TRAUMATOLOGY	19	2021	1.8	15.8
57	Kwong et al.	Posterior Urethral Valves Outcomes Prediction (PUVOP): a machine learning tool to predict clinically relevant outcomes in boys with posterior urethral valves	PEDIATRIC NEPHROLOGY	19	2022	2.6	10.5
58	Diller et al.	Denoising and artefact removal for transthoracic echocardiographic imaging in congenital heart disease: utility of diagnosis specific deep learning algorithms	INTERNATIONAL JOURNAL OF CARDIOVASCULAR IMAGING	19	2019	1.5	26.3
59	Blazadonakis et al.	Deep assessment of machine learning techniques using patient treatment in acute abdominal pain in children	ARTIFICIAL INTELLIGENCE IN MEDICINE	19	1996	6.1	147.4
60	Qu et al.	Using Innovative Machine Learning Methods to Screen and Identify Predictors of Congenital Heart Diseases	FRONTIERS IN CARDIOVASCULAR MEDICINE	18	2022	2.8	11.1
61	Wang et al.	Application of deep learning upon spinal radiographs to predict progression in adolescent idiopathic scoliosis at first clinic visit	ECLINICALMEDICINE	18	2021	9.6	16.7
62	Chang et al.	Improving preoperative risk-of-death prediction in surgery congenital heart defects using artificial intelligence model: A pilot study	PLOS ONE	18	2020	2.9	22.2
63	Jalali et al.	Prediction of Periventricular Leukomalacia in Neonates after Cardiac Surgery Using Machine Learning Algorithms	JOURNAL OF MEDICAL SYSTEMS	18	2018	3.5	33.3
64	Fernandez et al.	Digital Pattern Recognition for the Identification and Classification of Hypospadias Using Artificial Intelligence vs. Experienced Pediatric Urologist	UROLOGY	17	2021	2.1	17.6
65	Hoodbhoy et al.	Diagnostic Accuracy of Machine Learning Models to Identify Congenital Heart Disease: A Meta-Analysis	FRONTIERS IN ARTIFICIAL INTELLIGENCE	17	2021	3	17.6
66	Rani et al.	Predicting congenital heart disease using machine learning techniques	JOURNAL OF DISCRETE MATHEMATICAL SCIENCES & CRYPTOGRAPHY	17	2020	1.2	23.5
67	Thomford et al.	Implementing Artificial Intelligence and Digital Health in Resource-Limited Settings? Top 10 Lessons We Learned in Congenital Heart Defects and Cardiology	OMICS-A JOURNAL OF INTEGRATIVE BIOLOGY	17	2020	2.2	23.5
68	Nagy et al.	A pediatric wrist trauma x-ray dataset (GRAZPEDWRI-DX) for machine learning	SCIENTIFIC DATA	16	2022	5.8	12.5
69	Liu et al.	Incorporating Radiomics into Machine Learning Models to Predict Outcomes of Neuroblastoma	JOURNAL OF DIGITAL IMAGING	16	2022	2.9	12.5
70	Cirillo et al.	Improving burn depth assessment for pediatric scalds by AI based on semantic segmentation of polarized light photography images	BURNS	16	2021	3.2	18.8
71	Pasha et al.	Machine Learning Predicts the 3D Outcomes of Adolescent Idiopathic Scoliosis Surgery Using Patient-Surgeon Specific Parameters	SPINE	16	2021	2.7	18.8
72	Peng et al.	Prediction of Proximal Junctional Kyphosis After Posterior Scoliosis Surgery With Machine Learning in the Lenke 5 Adolescent Idiopathic Scoliosis Patient	FRONTIERS IN BIOENGINEERING AND BIOTECHNOLOGY	16	2020	4.3	25.0
73	Yin et al.	Multi-instance Deep Learning of Ultrasound Imaging Data for Pattern Classification of Congenital Abnormalities of the Kidney and Urinary Tract in Children	UROLOGY	16	2020	2.1	25.0
74	Malek et al.	Random forest and Self Organizing Maps application for analysis of pediatric fracture healing time of the lower limb	NEUROCOMPUTING	16	2018	5.5	37.5
75	Steyaert et al.	Multimodal deep learning to predict prognosis in adult and pediatric brain tumors	COMMUNICATIONS MEDICINE	15	2023	5.4	6.7
76	Zech et al.	Detecting pediatric wrist fractures using deep-learning-based object detection	PEDIATRIC RADIOLOGY	15	2023	2.1	6.7
77	Zhang et al.	Diagnostic Accuracy of 3D Ultrasound and Artificial Intelligence for Detection of Pediatric Wrist Injuries	CHILDREN-BASEL	15	2021	2	20.0
78	Koller et al.	Accurate prediction of spontaneous lumbar curve correction following posterior selective thoracic fusion in adolescent idiopathic scoliosis using logistic regression models and clinical rationale	EUROPEAN SPINE JOURNAL	15	2019	2.6	33.3
79	Maggio et al.	Distillation of the clinical algorithm improves prognosis by multi-task deep learning in high-risk Neuroblastoma	PLOS ONE	15	2018	2.9	40.0
80	Das et al.	Exercise capacity in pediatric heart transplant candidates: Is there any role for the 14 ml/kg/min guideline?	PEDIATRIC CARDIOLOGY	14	2006	1.5	128.6
81	Xiao et al.	Revolutionizing Healthcare with ChatGPT: An Early Exploration of an AI Language Model's Impact on Medicine at Large and its Role in Pediatric Surgery	JOURNAL OF PEDIATRIC SURGERY	14	2023	2.4	7.1
82	Shahi et al.	Decision-making in pediatric blunt solid organ injury: deep learning approach to predict massive transfusion, need for operative management, and mortality risk	JOURNAL OF PEDIATRIC SURGERY	14	2021	2.4	21.4
83	Stiel et al.	The Modified Heidelberg and the AI Appendicitis Score Are Superior to Current Scores in Predicting Appendicitis in Children: A Two-Center Cohort Study	FRONTIERS IN PEDIATRICS	14	2020	2.1	28.6
84	Van den Eynde et al.	Artificial intelligence in pediatric cardiology: taking baby steps in the big world of data	CURRENT OPINION IN CARDIOLOGY	13	2022	2	15.4
85	Mohsin et al.	The Role of Artificial Intelligence in Prediction, Risk Stratification, and Personalized Treatment Planning for Congenital Heart Diseases	CUREUS JOURNAL OF MEDICAL SCIENCE	13	2023	1	7.7
86	Sethi et al.	Artificial Intelligence in Pediatric Cardiology: A Scoping Review	JOURNAL OF CLINICAL MEDICINE	13	2022	3	15.4
87	Xu et al.	A Deep-Learning Aided Diagnostic System in Assessing Developmental Dysplasia of the Hip on Pediatric Pelvic Radiographs	FRONTIERS IN PEDIATRICS	13	2022	2.1	15.4
88	Feng et al.	Prediction for Mitosis-Karyorrhexis Index Status of Pediatric Neuroblastoma via Machine Learning Based 18F-FDG PET/CT Radiomics	DIAGNOSTICS	13	2022	3	15.4
89	Wang et al.	Characteristics of Fecal Microbiota and Machine Learning Strategy for Fecal Invasive Biomarkers in Pediatric Inflammatory Bowel Disease	FRONTIERS IN CELLULAR AND INFECTION MICROBIOLOGY	13	2021	4.6	23.1
90	van den Eynde et al.	Medicine-Based Evidence in Congenital Heart Disease: How Artificial Intelligence Can Guide Treatment Decisions for Individual Patients	FRONTIERS IN CARDIOVASCULAR MEDICINE	13	2021	2.8	23.1
91	Gómez-Quintana et al.	A Framework for AI-Assisted Detection of Patent Ductus Arteriosus from Neonatal Phonocardiogram	HEALTHCARE	13	2021	2.4	23.1
92	Bertsimas et al.	Prediction of cervical spine injury in young pediatric patients: an optimal trees artificial intelligence approach	JOURNAL OF PEDIATRIC SURGERY	13	2019	2.4	38.5
93	Haldar et al.	Unsupervised machine learning using K-means identifies radiomic subgroups of pediatric low-grade gliomas that correlate with key molecular markers	NEOPLASIA	12	2023	6.3	8.3
94	Amodeo et al.	A maChine and deep Learning Approach to predict pulmoNary hyperteNsIon in newbornS with congenital diaphragmatic Hernia (CLANNISH): Protocol for a retrospective study	PLOS ONE	12	2021	2.9	25.0
95	Gao et al.	Multimodal AI System for the Rapid Diagnosis and Surgical Prediction of Necrotizing Enterocolitis	IEEE ACCESS	12	2021	3.4	25.0
96	Nurmaini et al.	Deep Learning for Improving the Effectiveness of Routine Prenatal Screening for Major Congenital Heart Diseases	JOURNAL OF CLINICAL MEDICINE	11	2022	3	18.2
97	Herz et al.	Segmentation of Tricuspid Valve Leaflets From Transthoracic 3D Echocardiograms of Children With Hypoplastic Left Heart Syndrome Using Deep Learning	FRONTIERS IN CARDIOVASCULAR MEDICINE	11	2021	2.8	27.3
98	Kwon et al.	Deep learning algorithms for detecting and visualising intussusception on plain abdominal radiography in children: a retrospective multicenter study	SCIENTIFIC REPORTS	11	2020	3.8	36.4
99	Kim et al.	Performance of deep learning-based algorithm for detection of ileocolic intussusception on abdominal radiographs of young children	SCIENTIFIC REPORTS	11	2019	3.8	45.5
100	Liu et al.	Mining patient-specific and contextual data with machine learning technologies to predict cancellation of children's surgery	INTERNATIONAL JOURNAL OF MEDICAL INFORMATICS	11	2019	3.7	45.5

### 3.2 Analysis of countries and organizations

For the comprehensive and detailed analysis of contributing countries and organizations to these top 100 cited publications, we took the “individual authorship strategy” for counting countries or organizations. Briefly, if two authors from one paper came from the same country or organization, we counted the number of contributions about this country or organization as two rather than one.

**Figure 3:**
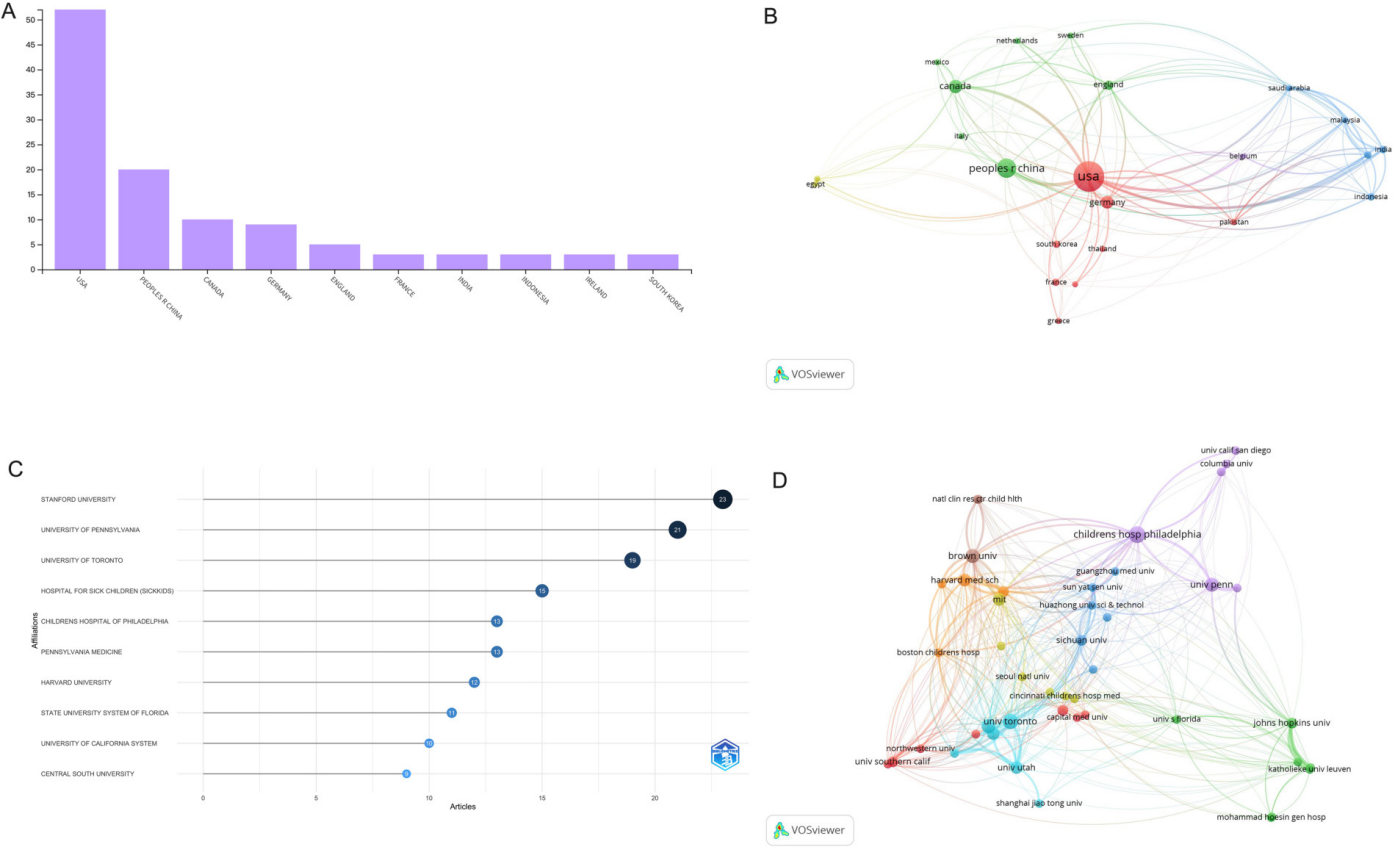
**(A)** Top 10 authorship countries by the number of publications. **(B)** Bibliographic coupling analysis of country-wise collaboration. **(C)** Top 10 authorship organizations by the number of publications. **(D)** Bibliographic coupling analysis of organization collaboration network.

A total of 41 countries have contributed related articles in the list. Of these, the USA dominated the global publication pattern (*n* = 52) (Figure 3A) as well as the leading position in country-wise collaboration, with the most total link strength (*n* = 2,552) (Figure 3B). It is pivotal to emphasize the broad country-wise collaboration in scientific work. Notable partnerships involved close collaborations among the USA, China and Germany (Figure 3B). Notwithstanding these active cooperations, there was a noticeable imbalance in different countries regarding research collaboration.

There were 304 organizations contributing to the 100 most cited manuscripts. The Stanford University dominated the publication number position (*n* = 23) (Figure 3C). While for the organization level collaboration, Children's Hospital of Philadelphia had the highest total link strength (*n* = 567) (Figure 3D).

### 3.3 Analysis of authors and journals

Similar to the “individual authorship strategy” for counting countries or organizations we applied in the analysis of countries and organizations. Here, for example, if one author played as both first and corresponding author, we counted this author' authorship as two rather than one.

**Figure 4:**
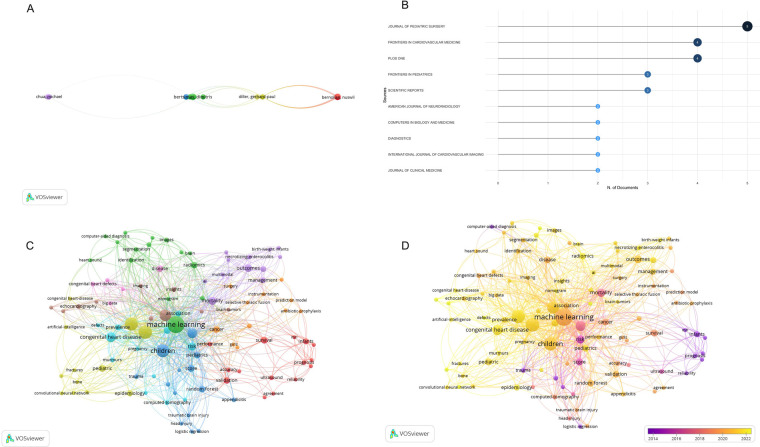
**(A)** Co-authorship collaboration network of authors. **(B)** Top 10 journals by the number of publications. **(C)** Mapping on co-occurrence of keywords for the top 100 cited articles. **(D)** Time visualization for the keywords. Keywords in yellow appeared later than that in purple.

Bertsimas D. from Massachusetts Institute of Technology held the leading position for the number of publications (*n* = 3) in the top 100 cited list. While for the international collaboration network analysis for authors, Arafati A. from University of California owned the highest total link strength (*n* = 498) (Figure 4A). However, from the collaboration analysis, we can find collaborations from authors in different organizations and countries were still not enough and should be enhanced.

There were 77 journals contributing to the top 100 cited papers. Of these, the *Journal of Pediatric Surgery* dominated the number of studies (*n* = 5; IF = 2.4), followed by the *Frontiers in Cardiovascular Medicine* (*n* = 4; IF = 2.8) and *PLOS ONE* (*n* = 4; IF = 2.9) (Figure 4B).

### 3.4 Keyword co-occurrence analysis

From keyword network visualization (Figure 4C) and overplay visualization (Figure 4D) based on VOSviewer, we might identify the current hotspots and future trends of artificial intelligence application in pediatric surgery, for a better understanding of the development of research key points. Our study contained a total of 560 all keywords, and there were 106 keywords with a frequency of more than or equal 2 times. The size of the colored nodes stands for the frequency of keyword occurrence, indicating the focus within the area. The linking lines between nodes represent the strength of connection, with thicker lines demonstrating more often co-appearance in one article. This network visualization promoted the recognition of eminent topics and associations in all keywords. Figure 4C depicted notable high-frequency keywords, including machine learning (ML), AI, DL, children, CHD and so on. When focusing on the emerging research cores in this field, we found necrotizing enterocolitis, CHD and radiomics may dominate potential hotspots in the future (Figure 4D).

## 4 Discussion

With the advent of digital time, it is crucial for scholars to entirely grasp the development in their research fields. Our study used a bibliometric method to investigate the present status and future trends of AI application in pediatric surgery. Unlike systematic review or meta-analysis, the bibliometric method applies visual software such as VOSviewer or bibliometrix package from Rstudio to analyze current publications in detail, in order to reveal research focus and predict trends.

For these 100 impactful publications in this field. The annual article production has presented a general rising trend from 1995 to 2023. Remarkably, the period between 2018 and 2021 witnessed a pivotal boost in AI technologies, including DL, ML and ChatGPT. This technological growth has provided extraordinary opportunities for the diagnosis, treatment and prediction of patients. These findings indicated the enhanced focus and interests from academia on AI application in pediatric surgery research, which was analogous to several other research fields (18, 19).

The USA stood out in the position for the number of articles, which was similar with other medical areas such as robotic arthroplasty and esophageal atresia (14, 20). This phenomenon is probably owning to the advanced technology and robust national support in the USA. In addition, previous literature has suggested that authors from the USA prefer to publish and cite native sources (21, 22). Remarkably, the government of USA issued an executive order titled “Maintaining American Leadership in Artificial Intelligence” mandating all federal government agencies to execute strategic goals for keeping the leading position in AI field on February 11, 2019 (18). Although China started later in AI field, it has become the second most productive country in the world, establishing close collaboration with the USA and Germany based on our country-wise collaboration map. Seven out of the top 10 publishing organizations were from the USA, two were from Canada and the other one was from China, which also indicated the dominant role of the USA in AI. Several findings can be drawn from the organization and country collaboration network in our study. The USA primarily collaborated with China and Germany, while other European countries tended to cooperate more with European Union countries. The USA and China owned both high scientific production and efficient collaboration, while other countries such as India, Indonesia and South Korea maintained considerable scientific outputs with relatively poor global cooperation. The challenges of global collaboration, such as time, cost, and integration, are likely responsible for this. Yet, there are mutual advantages to promote global collaborative efforts, including wider patient recruitment, better generalizability of results, scientific progress, and greater citation impact (23, 24). Collaboration at the global level should take place through international partnerships that lead and encourage action on health concerns by scheduling, support, and technical assistance (25). Due to the centrality of Bertsimas D. from Massachusetts Institute of Technology and Arafati A. from University of California, they were placed as the top authors in publication and worldwide collaboration field respectively. Studying their scientific outputs would be beneficial to understand of the knowledge structure in this area. The *Journal of Pediatric Surgery* dominated a higher publication count than other journals. Although the IF of the *Journal of Pediatric Surgery* is not the highest, due to it is one of the few journals which focus on pediatric surgery specifically, it contributed the highest number of publications. Researchers concentrating on AI application in pediatric surgery might pay more attention to this source.

Information analysis for the impactful articles is an advantageous index for bibliometric study, which is broadly utilized in various subjects (26, 27). The most cited article in the present analysis was published in 1995 by Peterson et al. in *Journal of Bone and Joint Surgery-American Volume*, predicting the progression of the curve in girls who had adolescent idiopathic scoliosis of moderate severity by using logistic regression analysis (16). The paper with the lowest impact index was released in 2019 by Hauptmann A et al. in the journal *Magnetic Resonance in Medicine*, investigating the potential of deep learning (DL) to reconstruct highly accelerated radial real-time data in patients with congenital heart disease (CHD) (17).

In the field of AI application in pediatric surgery, ML, AI, DL, children and CHD were identified highly frequent mentioned keywords according to the co-occurrence visualization analysis. ML and DL, as subsets of AI, they are especially effective for distinguishing subtle patterns among huge files that is probably imperceptible to humans using conventional statistical methods conducting manual investigations. They have been widely applied to analyze patient data for predicting outcomes and recovery times after surgery, the aim is to provide a better patient care as well as enhance the precision and efficacy of surgical procedures furtherly (28, 29). Notably, CHD was a hotspot in both current research and future trends, its prenatal diagnosis and postnatal management has progressed considerably with the AI assistance (30). Notwithstanding many advantages including precise prenatal screening, improved perioperative planning, acceptable individualized risk stratification and prognostication, a notable challenge for using AI in CHD has been the disconnect between clinical investigators and computer engineers (31). Therefore, computer engineers are necessary to be more familiar with clinical practice. In the meantime, clinicians lack of experience in AI filed shall get more information to better understand how AI works in medicine.

The level of evidence for a certain paper is probably an excellent index to evaluate its scientific quality (32). In the top 100 articles with the highest number of citations on AI application in pediatric surgery, publications with high evidence levels (level I and II), i.e., meta-analysis and prospective study, were underrepresented. For assessing the CHD diagnostic accuracy of ML models, one meta-analysis included 16 studies on 1,217 participants used ML algorithm to diagnose CHD, reporting ML models owned the potential to diagnose CHD correctly without the requirement for experienced personnel. However, the heterogeneity of the CHD diagnosis among these 16 studies was hard to ignore, which was a main limitation (33). One RCT used data from 964 pediatric patients with minor traumatic brain injury to compare the prediction function between ML and a nomogram, concluding that ML had the superior predictive performance which can assist clinicians with reducing the overuse of head CT scans and therapy costs of pediatric traumatic brain injury (34). The majority of the top 100 cited manuscripts were retrospective studies, which was vital in making research on manifestations and results for diverse cases. Nevertheless, their academic quality is limited: Several information might be lacking, selection and recall deviations can influence the outcomes, and reasons for differences in therapy procedure or inadequate follow-ups cannot be confirmed usually (35). Hence, to enhance the development of AI application in pediatric surgery, prospective studies and RCTs, as the superior standard of scientific work, are needed to a further step.

## 5 Limitations

Inevitably, there are still several limitations in the present study. Firstly, only the WoSCC database was applied to search for relevant studies, therefore, other sources such as Google Scholar or PubMed might have presented a different number of publication items or citations. To improve the comprehensiveness and representativeness of the analysis, we plan to include more data sources in future research. Secondly, only particular papers (English writing, article or review) were included, which may neglect several outstanding literatures published in different languages and cause biased outcomes. Thirdly, we aimed to obtain only related articles on AI application in pediatric surgery, thus the searching strategy of “title” instead of “topic” was utilized. This strategy could probably exclude few, but an insignificant number of pertinent literatures.

## 6 Conclusions

In conclusion, the present study performs the first comprehensive bibliometric analysis of impactful articles pertaining to AI application in pediatric surgery from 1995 to 2023. The USA and China lead the research frontiers, providing valuable opportunities for global cooperations. However, collaborations among developing countries need to be strengthen intensely. Necrotizing enterocolitis, CHD and radiomics might dominate potential hotspots in the future. This study may play a helpful role for researchers studying on AI application in pediatric surgery by providing insights into potential collaboration and prospects for future research.

## Data Availability

The original contributions presented in the study are included in the article/Supplementary Material, further inquiries can be directed to the corresponding author.
